# The association between climate variables and tuberculosis in Kolaka District, Southeast Sulawesi Province, Indonesia, 2013–2020: a Bayesian autoregressive model

**DOI:** 10.12688/f1000research.138859.2

**Published:** 2024-06-21

**Authors:** Ramadhan Tosepu, Asrul Sani, Devi Savitri Effendy, La Ode Ali Imran Ahmad

**Affiliations:** 1 Department of Environmental Health, Faculty of Public Health, Halu Oleo University, Kendari, South East Sulawesi, 90232, Indonesia; 2 Mathematics Department, Faculty of Mathematics and Sciences, Halu Oleo University, Kendari, South East Sulawesi, 90232, Indonesia; 3 Faculty of Public Health, Halu Oleo University, Kendari, South East Sulawesi, 90232, Indonesia

**Keywords:** Tuberculosis, Humidity, Temperature, Rainfall, Light, Indonesia

## Abstract

**Background:**

Tuberculosis is one of the diseases that requires comprehensive treatment. This disease is highly contagious and can be transmitted through the air. Climate factors play a role in the increasing cases of tuberculosis. This study aimed to determine the correlation between climatic variables and TB in Kolaka District, Southeast Sulawesi Province, Indonesia,

**Methods:**

This research was modeled using an autoregressive (AR) Bayesian model with three possible likelihoods; Gaussian, Poisson and Negative Binomial responses.

**Results:**

Minimum temperature and average temperature, a coefficient of 4.234 suggests that for every 1 degree increase in minimum temperature, there is an estimated increase of approximately four cases, assuming other variables remain constant. Maximum temperature, a coefficient of 17.851 suggests that for every 1 degree increase in maximum temperature, there is an estimated increase of around 17-18 cases, assuming other variables remain constant. Humidity, a coefficient of -13.413 suggests that for every 1% increase in humidity, there is an estimated decrease of around 13 cases, assuming other variables remain constant. Rainfall, a coefficient of -0.327 suggests that for every 1 mm increase in rainfall, there is an estimated decrease of around 0.327 cases, assuming other variables remain constant. Light, a coefficient of -4.322 suggests that for every 1-hour increase in light duration, there is an estimated decrease of around four cases, assuming other variables remain constant.

**Conclusions:**

Climate change has a significant impact on tuberculosis through temperature-related factors. These factors influence the prevalence, spread, and vulnerability to TB. Addressing these challenges requires a holistic approach involving adaptation planning. Strong public health systems and healthcare infrastructure can help mitigate the risks and impacts of climate change-related tuberculosis.

## Introduction

Tuberculosis is an infectious disease caused by the bacterium *Mycobacterium tuberculosis* (MTB). ^1^ This disease primarily affects the respiratory system, particularly the lungs, but it can also attack other organs in the body. ^2^^,^^3^ TB has become one of the deadliest diseases in the world, with millions of people infected and thousands of deaths occurring annually. Globally, there are 6.4 million cases of tuberculosis, which accounts for 64% of tuberculosis incidents. tuberculosis still remains among the top 10 causes of death worldwide, with an estimated 1.3 million tuberculosis related deaths globally. ^4^ In Indonesia, TB still remains one of the diseases with the highest number of cases, requiring continued attention from the government.

According to the Global Tuberculosis Report 2017, it is estimated that there were 1.020.000 tuberculosis cases in Indonesia, ranking second in the world for the highest number of TB cases after India. The number of new tuberculosis cases recorded in Indonesia in 2017 was 420.994. ^5^ According to the Tuberculosis Data and Information Center of the Ministry of Health of the Republic of Indonesia in 2017, the indicators used for evaluating and monitoring the success of tuberculosis control efforts are the detection of new tuberculosis cases (case detection rate (CDR)) and the treatment success rate. These indicators serve as important measures to assess the progress and effectiveness of tuberculosis management in Indonesia.

The tuberculosis cases in Indonesia are generally high and rapidly growing among individuals aged 15 years and above, people living in poverty, those with low educational levels, and socioeconomically disadvantaged communities. West Java is the province that contributes the highest number of cases in Indonesia, with the majority occurring among individuals aged 15 years and above. ^6^ Climate change has become an increasingly urgent global issue in the past few decades. ^7^ In recent years, the impact of climate change on human health has been a major concern for scientists and health experts worldwide. ^8^ One significant aspect of health that is significantly affected by climate change is the spread of infectious diseases, including TB. ^9^^,^^10^

Climate change and the spread of TB involve various complex factors. ^11^ One key factor is the changes in temperature and rainfall patterns that can affect the environment where TB bacteria can survive and multiply. ^12^ Increased average temperatures in certain regions can lead to changes in the ecology of vectors that act as disease carriers. ^13^^–^^15^ Additionally, changes in rainfall patterns can affect the availability of clean water, which is crucial for ensuring adequate cleanliness and sanitation, both of which are important factors in TB prevention. ^16^^,^^17^

In Brazil, it has been found that tuberculosis cases are more frequent under the following conditions: ultraviolet radiation (UVR) above 17 MJ/m ^2^ (67.8%; *p* ≤ 0.001); relative humidity between 31.0% and 69.0% (95.8%; *p* ≤ 0.001); rainfall less than 1 mm (71.7%; *p* ≤ 0.001); daily sun exposure for 12 hours (40.6%; *p* = 0.001); and temperatures between 20°C and 23°C (72.4%; *p* ≤ 0.001). ^18^ It has been found that extremely high temperatures have a positive effect on the reported morbidity of pulmonary tuberculosis in Binzhou (RR = 0.924, 95% CI: 0.856–0.997) and Weihai (RR = 0.910, 95% CI: 0.843–0.982). ^19^ In Hong Kong, it has also been found that lower average temperatures (<22.0°C) in the later months (>10 months) are associated with an increased risk of tuberculosis notifications. Specifically, the risk increases within the temperature range of 16.3 to 17.3°C at lag 13-15 months and reaches the highest risk at a temperature of 16.8°C at lag 14 months. ^20^ According to Zhang *et al*. (2019) using a Geographically Weighted Regression (GWR) model, a positive correlation was found between TB incidence and annual average rainfall (AR), but a negative correlation was observed with other meteorological factors. Average relative humidity (ARH) also showed a negative correlation with TB incidence in all prefectures with a significance level of *p* < 0.05. ^21^

Humidity is indeed a climate factor that can potentially affect the spread of tuberculosis. ^22^^,^^23^ Several studies have shown that high humidity can support the survival of MTB bacteria in the environment. ^24^^,^^25^ High humidity can also influence the dispersal of respiratory droplets containing MTB bacteria. ^26^^,^^27^ Under low humidity conditions, respiratory droplets can dry more quickly, and the bacteria-containing particles become lighter, enabling them to remain suspended in the air for longer periods. ^28^^,^^29^ Therefore, low humidity can also increase the risk of tuberculosis transmission.

Indeed, climate change can also have an impact on the overall human health system. Fluctuations in extreme temperatures, extreme weather events such as floods or droughts, and changes in seasonal patterns can cause social and economic instability, which in turn can affect the spread of TB. These factors can lead to human migration, food insecurity, reduced nutritional status, and vulnerability to infectious diseases, including TB.

## Methods

### Location of study

Astronomically, Kolaka Regency is located along the equator section of the equator, extending from North to South between 3°36′ - 4°35′ South Latitude (SL) and extending from West to East between 120° 45′-121° 52′ East Longitude (EL). Based on its geographical position, the territorial boundaries of Kolaka Regency in the north is Kolaka Utara Regency, in the south is Bombana regency, in the east is Kolaka Timur Regency and in the west is Sulawesi Selatan Province in Teluk Bone.

### Date sources

This study utilized climate data and tuberculosis disease data. The climate data were obtained from the Meteorology and Geophysics Agency of Southeast Sulawesi Province, including temperature, minimum temperature, maximum temperature, humidity, rainfall, and light data from 2013 to 2020. The data are accessible on the DATA ONLINE - PUSAT DATABASE – BMKG website. Meanwhile, the tuberculosis data were obtained through reports from the Health Department of Kolaka District from 2013 to 2020. The data on tuberculosis disease originates from the health profile of Kolaka district. This data can be accessed openly through the Kolaka District website.

### Statistical analysis

The association between meteorological factors and the number of TB cases was modeled using an autoregressive (AR) Bayesian model with three possible likelihoods; Gaussian, Poisson and Negative Binomial responses. Although TB cases are in fact discrete as both the Poisson and NB distributions, the continuous Gaussian distribution is also suitable to use as the number of TB cases is large. In case the dispersion statistic is close to one, a Poisson model is better to apply than NB distribution. In this study, however, we use both WAIC and DIC values as the model selection criterion to evaluate their performance.

Suppose $y_{t}$ denote the monthly/annual counts of TB in the District of Kolaka, t=1,2,…,8 representing the period of 2013-2020 and $u_{t}$ represents meteorological covariates; i.e., minimum temperature, maximum temperature, average temperature, humidity, rainfall and light. Then, the response $y_{t}$ follows one of three response likelihoods;

For Gaussian distribution with mean $\alpha +u_{t}$ ;

$$y_{t}=\alpha +u_{t}+\varepsilon_{t}.$$

For Poisson distribution;

$$y_{t}∼\text{Pois}\left(\mu_{t}\right),t=1,\ldots ,T$$

$$\log\left(\mu_{t}\right)=\alpha +u_{t},t=1,\ldots ,T$$

For Negative Binomial (NB) distribution

$$y_{t}∼\mathrm{NB}\left(\mu_{t}\right),t=1,\ldots ,T$$

$$\log\left(\mu_{t}\right)=\alpha +u_{t},t=1,\ldots ,T$$

The term $u_{t}$ represents meteorological covariates; *i.e.*, minimum temperature, maximum temperature, average temperature, humidity, rainfall and light.

Then, the temporal model is formulated as autoregressive order 1, which reads

$$u_{1}∼N\left(0,\tau_{u}{\left(1-\rho^{2}\right)}^{-1}\right)$$

$$u_{t}=\rho u_{t-1}+\varepsilon_{t},t=2,\ldots ,T$$

$$\varepsilon_{t}∼N\left(0,\tau_{u}\right),t=1,\ldots ,T$$

where α is an intercept, ρ is a temporal correlation term (with $\left|\rho \right|<1$ ) and $\varepsilon_{t}$ is a Gaussian error term with zero mean and a fixed large precision $\tau_{u}$ so that the error term is close to zero.

The performance of all models on the TB data in the district of Kolaka, Southeast Sulawesi from 2013 to 2020, based on the DIC (WAIC) values, is summarized in Table 1.

**Table 1.** The performance of autoregressive model with three likelihoods based on the values.

Gaussian	Poisson	Negative Binomial
101.7687 (102.4905)	76.50949 (74.06891)	93.56521 (93.98121)

Table 1 shows that the performances of the autoregressive model with Poisson likelihood is much better, compared to those with Negative Binomial and Gaussian responses. This indicates that Poisson is suitable for analyzing tuberculosis data. Therefore, using Poisson autoregressive model, we determined the influence of meteorological factors on TB incidence in the district of Kolaka, Southeast Sulawesi from 2013 to 2020.

### Ethical considerations

The study obtained ethical approval from the Research Ethics Committee of the Indonesian Public Health Association (IPHA) Southeast Sulawesi Province under approval number 134/KEPK-IAKMI/II/2023.

## Results

Table 2 ^73^ shows that the linear regression model conducted to test the correlation between climate factors and tuberculosis disease resulted in values above 0.05, indicating that there was no significant influence of each variable. Furthermore, a test using the Bayesian autoregressive model will be conducted to further investigate the relationship.

**Table 2.** Data analysis utilizing a linear regression model.

Variables	Estimate	Std. Error	*t value*	*Pr(>|t|)*
(Intercept)	-4932.2035	1525.0405	-3.2341	0.1909
Temp.Min	398.4906	98.1125	4.0616	0.1537
Temp.Max	-124.1586	50.3234	-2.4672	0.2451
Temp.Avg	785.0713	177.6283	4.4197	0.1417
Humidity	-261.3844	53.6304	-4.8738	0.1288
Rainfall	5.7134	1.4102	4.0514	0.1541
Light	-35.7148	7.4783	-4.7758	0.1314

Table 3 indicates that the minimum temperature, maximum temperature, and average temperature have positive values of one, which are quite high, indicating a positive association with TB cases. On the other hand, humidity, rainfall, and light have negative values, indicating a negative association with tuberculosis cases. Further analysis of the coefficient model revealed the following relationships: i) minimum temperature and average temperature, a coefficient of 4.234 suggests that for every 1 degree increase in minimum temperature, there is an estimated increase of approximately four cases, assuming other variables remain constant. ii) Maximum temperature, a coefficient of 17.851 suggests that for every 1 degree increase in maximum temperature, there is an estimated increase of around 17-18 cases, assuming other variables remain constant. iii) Humidity, a coefficient of -13.413 suggests that for every 1% increase in humidity, there is an estimated decrease of around 13 cases, assuming other variables remain constant. iv) Rainfall, a coefficient of -0.327 suggests that for every 1 mm increase in rainfall, there is an estimated decrease of around 0.327 cases, assuming other variables remain constant. v) Light, a coefficient of -4.322 suggests that for every 1-hour increase in light duration, there is an estimated decrease of around four cases, assuming other variables remain constant.

**Table 3.** Data analysis employing a Bayesian autoregressive model.

Variables	mean	sd	0.025quant	0.5quant	0.975quant	mode
(Intercept)	883.084	1641.260	-2389.422	879.143	4168.313	867.640
Temp.Min	4.234	29.597	54.065	4.291	62.165	4.410
Temp.Max	17.851	27.344	-37.218	18.337	70.257	19.364
Temp.Avg	4.234	30.259	-55.264	4.263	63.517	4.324
Humidity	-13.413	17.136	-45.864	-13.897	21.823	-14.830
Rainfall	-0.327	0.832	-2.024	-0.312	1.284	-0.284
Light	-4.322	8.207	-20.147	-4.527	12.696	-4.895

## Discussion

In recent decades, climate change has become a major global issue that has garnered significant attention. The wide-ranging impacts of climate change include increased global average temperatures, changes in rainfall patterns, increased drought occurrences, and heightened frequency and intensity of natural disasters. ^30^^–^^32^ Furthermore, human health is also significantly affected by climate change, with tuberculosis being one of the diseases impacted. ^33^^,^^34^ Our findings indicate that climate change is likely to affect individual vulnerability to tuberculosis by increasing the prevalence of underlying risk factors, particularly in developing countries.

Indeed, climatic conditions are one of the factors that can influence the development of disease-causing microorganisms. ^18^^,^^35^ Certain microorganisms, including bacteria, viruses, and fungi, can thrive or struggle depending on the environmental conditions they are exposed to. Temperature, humidity, precipitation, and other climatic factors can directly or indirectly affect the survival, growth, reproduction, and transmission of these microorganisms. ^36^^–^^38^ For example, warm and humid environments may promote the proliferation of certain bacteria and fungi, while extreme temperatures or drought conditions may inhibit their growth. Understanding the relationship between climate and the development of pathogenic microorganisms is crucial for assessing and addressing public health risks. ^39^ Climate change can lead to changes in ecosystems, altering the patterns of interaction between humans and the environment. These changes can have significant impacts on human health. For example, shifts in temperature and precipitation patterns can affect the distribution of disease vectors such as mosquitoes, ticks, and other carriers of infectious diseases. This can lead to the spread of vector-borne diseases like malaria, dengue fever, or Lyme disease into new regions or the prolongation of their transmission seasons. ^29^^,^^39^^–^^41^

Climate change can also contribute to the exacerbation of respiratory and cardiovascular diseases due to increased air pollution, the release of allergens, and the intensification of heatwaves. Moreover, it can have indirect effects on mental health and well-being, particularly among communities affected by climate-related disasters or forced migration. ^42^^,^^43^ Population density can increase the risk of increased exposure for individuals suffering from pulmonary tuberculosis, which in turn facilitates the spread of the bacteria causing it. ^44^^,^^45^ In addition to density factors, the occurrence of tuberculosis is also influenced by other risk factors such as high poverty levels, low coverage of healthy housing, and poor hygiene and health practices. Therefore, the high number of detected tuberculosis cases in a particular area may also be influenced by the characteristics of other variables. ^6^^,^^46^

Most bacteria and fungi are able to survive in air humidity reaching or exceeding 70%. ^47^ In conditions where the monthly average air humidity in Kabupaten Serang ranges from 70% to 87%, bacteria and fungi can thrive in the outdoor environment. The presence of a negative relationship between air humidity and the number of new tuberculosis cases may suggest that indoor air humidity is not associated with the presence of TB-causing agents inside the home. ^48^ This finding is supported by research conducted by Duffield and Young (1984), which demonstrated that pathogenic MTB bacteria can survive equally well in both damp and dry soil. ^49^

Global temperature increase can impact the ecology and geography of tuberculosis. ^18^^,^^50^ The role of climate in tuberculosis spread cannot be seen as a single factor. Temperature can influence the transmission of tuberculosis. The MTB bacteria can survive in various temperatures. Both low and high temperatures can affect the survival of the bacteria outside the human body. In specific areas, low temperatures can create more favorable conditions for the bacteria to survive outside the human body and trigger an increased risk of transmission. Global climate change can affect the patterns of tuberculosis spread. Seasons and local climates can also influence the occurrence of tuberculosis. Several studies indicate a relationship between specific seasons and increased tuberculosis cases. Factors such as temperature, air humidity, and rainfall patterns can influence the transmission rate and severity of the disease. Warmer and more humid temperatures can increase the bacteria’s survival rate in the environment, thereby complicating the control of disease transmission. ^42^^,^^51^^–^^53^

The research findings from the present study did not find any correlation between humidity and tuberculosis in Kolaka Regency. The influence of humidity on tuberculosis involves multiple factors. It should be noted that TB is a contagious disease most commonly transmitted through the air ( *via* infected airborne droplets), and other factors such as direct contact with untreated patients with tuberculosis or a weakened immune system also play a significant role in its transmission. ^17^^,^^41^^,^^54^

High relative humidity in indoor environments can affect the transmission of tuberculosis. When the relative humidity is above 80%, the risk of contracting tuberculosis significantly increases. High humidity in indoor spaces can impact the spread of tuberculosis by prolonging the survival time of infected airborne droplets in the air, thus allowing for a longer duration of inhalation and transmission. However, other studies have found a correlation between humidity and tuberculosis. Low humidity, especially during the dry season, is associated with an increased risk of tuberculosis infection. This occurs because low humidity can affect the respiratory tract and make individuals more vulnerable to tuberculosis infection. While there is some evidence indicating a relationship between humidity and tuberculosis transmission, further research is still needed to fully understand the interaction between humidity and this disease. ^20^^,^^22^^,^^55^^–^^57^

In this research, no correlation was found between rainfall and tuberculosis. However, during the period of 2013-2020, the presence of tuberculosis in Kolaka Regency tended to increase. Rainfall and tuberculosis are two different factors that can be related to each other. Rainfall refers to the amount of precipitation that falls in a specific period in a given area. High rainfall can create a humid environment. This humidity can affect the survival of MTB bacteria in the external environment. These bacteria are more resistant to moist conditions and can survive in airborne water droplets for a longer time. This means that in areas with high rainfall, these bacteria can remain active and easily spread through water droplets. Rainfall can also determine the amount of time hosts spend indoors, thus influencing the transmission of MTB within households. ^38^

Indeed, there are other factors that can contribute to the increase of tuberculosis. Areas with high rainfall tend to have a higher population density, and high population density can lead to poor environmental conditions. ^16^^,^^58^ Poor environmental conditions, such as high density, lack of ventilation, and poor sanitation, can worsen the spread of tuberculosis. People living in areas with high rainfall may be forced to reside in unhealthy environments, such as overcrowded settlements in urban areas that lack adequate sanitation facilities, which can increase the risk of TB transmission. Poor economic conditions can result in unemployment, poverty, and diverted funds, reducing access to proper healthcare. Social and economic instability can also affect individuals’ ability to seek care, adhere to appropriate treatment, and isolate themselves when infected with tuberculosis. ^59^^–^^62^

Furthermore, in Kolaka Regency, no relationship was found between sunlight exposure and the occurrence of tuberculosis. Indirect sunlight exposure can influence TB cases by affecting the host’s condition. Areas with limited sunlight exposure have been reported to have a higher prevalence of vitamin D deficiency. ^63^ Sunlight plays a crucial role in the production of vitamin D in the body. When the skin is exposed to sunlight, it synthesizes vitamin D, which is essential for maintaining healthy bones and overall health. Adequate sunlight exposure is necessary for the body to produce an optimal amount of vitamin D. ^64^ Absolutely, vitamin D deficiency can indeed affect the antimicrobial peptide system, which is responsible for regulating the immune response in the human body. ^65^^,^^66^ Vitamin D plays a crucial role in modulating the immune system and enhancing the function of antimicrobial peptides, which are essential for fighting against microbial infections. ^67^^,^^68^ Insufficient levels of vitamin D can compromise the immune system’s ability to defend against pathogens, including MTB, the bacteria responsible for TB. ^69^^,^^70^ Impaired host immunity due to vitamin D deficiency will support the development and reactivation of TB disease. ^71^

In addition, sunlight exposure can also affect the life cycle of TB-causing agents. MTB can survive in the environment for more than 74 days if protected from light. ^49^ MTB in sputum will die within a short period of time when exposed to sunlight, up to seven hours. ^18^ Therefore, access to sunlight inside buildings is crucial to prevent the formation of conducive environments for MTB within the house.

Changes in rainfall patterns and temperature can impact the life cycle of MTB bacteria and the transmission vectors of this disease. ^53^ High humidity and abundant rainfall can create ideal environmental conditions for the bacteria to survive and proliferate. ^52^ Climate change can affect population migration, whether caused by natural disasters or socioeconomic changes. ^51^ Migration can lead to the spread of TB to areas previously free from the disease. Migration can also disrupt access to adequate healthcare, worsen the health conditions of individuals infected with TB, and increase the risk of transmission. ^37^^,^^72^

To control the spread of tuberculosis, especially in high-risk areas, it is important to follow recommended preventive measures such as early treatment for patients with tuberculosis, ensuring good ventilation in rooms, maintaining personal and environmental hygiene, and promoting awareness about the importance of respiratory hygiene and healthy living practices. Research has limitations, as factors like population density, ventilation quality, direct exposure to untreated tuberculosis patients, and individual immune status also play significant roles in the spread of the disease. Therefore, future studies should consider examining these factors in order to gain a comprehensive understanding.

## Conclusions

Climate change has a significant impact on tuberculosis through temperature-related factors. These factors influence the prevalence, spread, and vulnerability to tuberculosis. Addressing these challenges requires a holistic approach involving adaptation planning. Strong public health systems and healthcare infrastructure can help mitigate the risks and impacts of climate change-related tuberculosis. Prevention efforts, early diagnosis, and effective treatment are crucial in controlling the spread of this disease. In the context of climate change, efforts to enhance capacity and resources are needed to address emerging challenges.

## Data Availability

Figshare: Climate and Tuberculosis data.xlsx. https://doi.org/10.6084/m9.figshare.24329518.v1. ^73^ Data are available under the terms of the Creative Commons Attribution 4.0 International license (CC-BY 4.0).
