# Supplementary material for: The association between climate variables and tuberculosis in Kolaka District, Southeast Sulawesi Province, Indonesia, 2013–2020: a Bayesian autoregressive model
Source: F1000Res. 2024 Jun 21;12:1507. Originally published 2023 Nov 27. [Version 2] doi: 10.12688/f1000research.138859.2 (PMC11339591; doi:10.12688/f1000research.138859.2)
Supplement: Peer review history

# Reviewer report: Reviewer response for version 2

**DOI:** 10.5256/f1000research.167785.r306628

**Published:** 2024-08-21

**Authors:** Paola Di Carlo

**Affiliations:** 1 University of Palermo, Palermo, Italy; 2 Department of Health Promotion, Mother and Child Care, Internal Medicine and Medical Specialties, University of Palermo, Palermo, 90127, Italy

Please take note of the following text:

 The manuscript, a significant contribution to our understanding of the relationship between climate change and infectious diseases, specifically tuberculosis, focuses on the authors' insightful statistical analysis. This analysis effectively connects climate variables with the prevalence of tuberculosis, potentially paving the way for impactful interventions and policies.

 It would be enhanced by including discussions or introductions about epidemiological geography, supported by figures, to make the manuscript more engaging. Geographical features and seasonality can significantly influence climatic variables and tuberculosis transmission.

 Please ensure that the following manuscript is cited in the references regarding geography, seasonal distribution, and climate change.

Is the work clearly and accurately presented and does it cite the current literature?

Partly

If applicable, is the statistical analysis and its interpretation appropriate?

Yes

Are all the source data underlying the results available to ensure full reproducibility?

Yes

Is the study design appropriate and is the work technically sound?

Yes

Are the conclusions drawn adequately supported by the results?

Yes

Are sufficient details of methods and analysis provided to allow replication by others?

Yes

Reviewer Expertise:

Climate change, antibiotic resistance, tuberculosis

I confirm that I have read this submission and believe that I have an appropriate level of expertise to confirm that it is of an acceptable scientific standard.

## References

[1] : The Impact of the Seasonal and Geographical Distribution of Tuberculosis in Sicily: A 6-Year Retrospective Study (2018-2023). *J Clin Med* .2024;13(12) : 10.3390/jcm13123546 10.3390/jcm13123546 38930075 PMC11204755

[2] : Tuberculosis evolution and climate change: How much work is ahead?. *Acta Trop* .2019;190: 10.1016/j.actatropica.2018.11.016 157-158 10.1016/j.actatropica.2018.11.016 30452890

# Reviewer report: Reviewer response for version 2

**DOI:** 10.5256/f1000research.167785.r294011

**Published:** 2024-06-24

**Authors:** Chacha M. Issarow

**Affiliations:** 1 Universiity of Cape Town, Cape Town, South Africa

I can confirm that the issues raised have been addressed by the authors and that the paper can be accepted.

Is the work clearly and accurately presented and does it cite the current literature?

Partly

If applicable, is the statistical analysis and its interpretation appropriate?

Partly

Are all the source data underlying the results available to ensure full reproducibility?

No

Is the study design appropriate and is the work technically sound?

Yes

Are the conclusions drawn adequately supported by the results?

Yes

Are sufficient details of methods and analysis provided to allow replication by others?

Partly

Reviewer Expertise:

Bioinformatics and infectious diseases mathematical modelling

I confirm that I have read this submission and believe that I have an appropriate level of expertise to confirm that it is of an acceptable scientific standard.

# Reviewer report: Reviewer response for version 1

**DOI:** 10.5256/f1000research.152089.r247572

**Published:** 2024-03-05

**Authors:** Chacha M. Issarow

**Affiliations:** 1 Universiity of Cape Town, Cape Town, South Africa

In this paper, the authors explore the association between climate change and TB transmission using a Bayesian autoregressive model. The paper has some merits as it highlights environmental factors attributable to climatic change that are correlated to TB transmission. I assessed all sections of the paper and provided some recommendations as outline below. The paper can be approved if those issues can be addressed by the authors.

**Introduction:**

1. The authors mentioned that according to the Global Tuberculosis Report 2017, it is estimated that there were 1.020.000 tuberculosis cases in Indonesia, ranking second in the world for the highest number of TB cases after India, and that the number of new tuberculosis cases recorded in Indonesia in 2017 was 420.994. I understand that the paper explored TB transmission in 2013–2020, but I would suggest adding a citation of the recent report because the TB prevalence could be high or low. Otherwise, this section was well explained about how factors related to climate change contribute to TB transmission.

**Methods:**

1. Methodology is one of the key parts of the paper, and it should be clearly explained so that other researchers can adopt or populate it in their studies. I would suggest the authors further explain the parameter μt to clarify the difference between the Poisson distribution and the negative binomial distribution, and how they are different from the Gaussian distribution.
2. Based on the model, it’s not clear how Table 1 was formed, and it would be worth it if it were clarified how those values were obtained and which data/value used to create the table.
3. It would be good to see some more critical discussion of the limitations of the model, and what experimental data would be useful to improve the model.

R **esults and abstract**

1. The author postulated that “minimum temperature, a coefficient of 4.234 suggests that for every 1 degree increase in minimum temperature, there is an estimated increase of approximately four cases, assuming other variables remain constant,"  and "average temperature, a coefficient of 4.234 suggests that for every 1 degree increase in average temperature, there is an estimated increase of approximately four cases, assuming other variables remain constant." These sentences sound like repetitions with the same value. I would suggest removing one of these sentences or connecting them (using minimum and average temperature) to one sentence to avoid repetition.

**Discussion:**

1. The authors mentioned that “additionally, changes in climate can impact water resources, food production, and the availability of clean air, all of which are essential for maintaining good health. Extreme weather events, such as hurricanes, floods, or heatwaves, can cause injuries, displacement, and even loss of life. Moreover, these events can also lead to the contamination of water sources, the destruction of infrastructure, and the disruption of healthcare services.” This paragraph has no direct link between climate change and TB. I would recommend removing this paragraph from the paper.

Is the work clearly and accurately presented and does it cite the current literature?

Partly

If applicable, is the statistical analysis and its interpretation appropriate?

Partly

Are all the source data underlying the results available to ensure full reproducibility?

No

Is the study design appropriate and is the work technically sound?

Yes

Are the conclusions drawn adequately supported by the results?

Yes

Are sufficient details of methods and analysis provided to allow replication by others?

Partly

Reviewer Expertise:

Bioinformatics and infectious diseases mathematical modelling

I confirm that I have read this submission and believe that I have an appropriate level of expertise to confirm that it is of an acceptable scientific standard, however I have significant reservations, as outlined above.
